# Comparison of Effectiveness of Two Different Doses of Propranolol on Kaposiform Hemangioendothelioma

**DOI:** 10.3389/fped.2022.760401

**Published:** 2022-03-28

**Authors:** Li Wei, Li Li, Zigang Xu, Bin Zhang, Xiaofeng Han, Chen Wang, Yuanxiang Liu, Bingyu Xiu, Lei Qiu, Yujuan Sun, Lin Ma

**Affiliations:** Department of Dermatology, National Center for Children's Health, Beijing Children's Hospital, Capital Medical University, Beijing, China

**Keywords:** kaposiform hemangioendothelioma, propranolol, dosage, course, safety

## Abstract

**Objective:**

To compare the clinical efficacy and safety of two different doses of propranolol in the treatment of cutaneous kaposiform hemangioendothelioma (KHE).

**Methods:**

The cohort of this prospective case–control study comprised 11 children with KHE treated from October 2015 to August 2018 in our institution. All participants were clinically and pathologically diagnosed as having cutaneous KHE. The children were allocated to two groups: six children in Group A (low-dose group) received oral propranolol 1.5 mg/kg/d, whereas five in Group B (high-dose group) received oral propranolol 2 mg/kg/d. The children were checked and photographed before and after treatment. Changes in the tumors were tracked by clinical and ultrasound examination. Follow-up visits to monitor for adverse reactions occurred regularly.

**Results:**

Grade I, Grade II, and Grade IV improvements in tumors were each noted in one child in Group A (three improved in total) and Grade III in two and Grade IV in another two children in Group B (four improved in total). Oral propranolol was effective in 50 and 80% of children in Groups A and B, respectively; this difference is statistically significant (*P* < 0.01). Minor adverse reactions occurred in eight of the 11 children.

**Conclusions:**

Propranolol treatment is effective against cutaneous KHE. There were no serious adverse reactions, and the treatment was safe in the long term. A dose of 2 mg/kg/d was more effective than 1.5 mg/kg/d in the treatment of KHE and did not increase the rate of adverse reactions. Children with KHE should be treated with propranolol 2 mg/kg/d orally.

## Introduction

Kaposiform hemangioendothelioma (KHE), a rare vascular tumor with lymphatic endothelial differentiation, characteristically occurs in infancy or early childhood (1, 2). It can cause Kasabach–Merritt phenomenon (KMP), which is characterized by severe thrombocytopenia and consumptive coagulation and is one of the most serious potentially fatal diseases in the neonatal period, seriously threatening the lives of young children.

Propranolol, a non-selective β-adrenergic receptor blocker, has been successfully used to treat infantile hemangioma, its use having gradually been extended to treating KHE. However, propranolol reportedly has different therapeutic effects on KHE (3, 4). A preliminary study has shown that propranolol is suitable for treating KHE in patients with small lesions and without KMP and that the higher the blood concentration of propranolol, the better the therapeutic effect.

Our aim was to investigate the use of propranolol to treat KHE. We drew on the reports of the treatment of infantile hemangioma and the characteristics of drug metabolism of propranolol in Chinese individuals to decide on two dosage levels, 1.5 mg/kg/d and 2 mg/kg/d. Our findings enabled us to establish the efficacy and optimal dosage of oral propranolol for treating KHE.

## Materials and Methods

### Participants

The study cohort comprised 11 children with KHE who attended our hospital from October 2015 to August 2018. The inclusion criteria were as follows: (1) diagnosed with KHE; (2) older than 28 days; (3) stable vital signs; (4) parents or legal guardians voluntarily gave their written informed consent; and (4) all clinical manifestations and imaging and pathological findings were consistent with the diagnostic criteria for KHE. All lesions were located in the skin of the participants, and none had KMP on enrollment. On histopathological examination, KHE was characterized by nodules of infiltrating spindled endothelial cells, dilated and hyperplastic lymphatic channels, and slit-like vascular channels. Typical immunohistochemistry findings included D2-40+, CD31+, CD34+, VEGFR-3+, and GLUT-1+. The exclusion criteria comprised of the following: (1) no contraindication to propranolol; (2) participating in other clinical research; and (3) researchers judged that compliance would be poor and the study requirements would not be fulfilled. All treatment plans were approved by the Ethics Research Association of our institution.

### Study Protocol

This was a randomized controlled study. After the parents had given their written informed consent, the children were randomly allocated into two groups: Group A, propranolol 1.5 mg/kg/d (six children) and Group B, propranolol 2 mg/kg/d (five children). All the children were admitted to our hospital to commence oral propranolol treatment. The initial dose was 0.375 mg/kg or 0.5 mg/kg twice a day. If all relevant indexes were stable, propranolol dosages were increased to 0.75 mg/kg or 1 mg/kg twice a day at 12-h intervals on the second day (Groups A and B, respectively). Blood pressure, blood glucose, heart rate, and general condition were monitored during treatment. Photos were taken before and after treatment. Investigations included liver and kidney function, myocardial zymography, tumor ultrasound, electrocardiogram, and cardiac color ultrasound. The children were followed up in accordance with the treatment protocol, including the evaluation of efficacy and adverse reactions. They were followed up once a month before commencing propranolol, then 1, 3, 6, and 12 months after commencing it. Follow-up was at 3-month intervals after the cessation of propranolol treatment.

### Evaluation of Efficacy

Efficacy was evaluated by visual analog scale scores and local B-ultrasound to determine changes in tumor size or thickness compared with before treatment. Responses were evaluated using the following four-grade classification, which was proposed by Achauer et al. (5): Grade I: tumor size reduced by 0–25% or its surface is lighter in color than before; Grade II: tumor size reduced by 26–50% or its surface is lighter in color; Grade III: tumor size reduced by 51–75% and its surface lighter in color; and Grade IV: tumor has shrunk by >75% or its surface color has completely faded away. The rate of effectiveness was calculated as the number of patients with Grade II to Grade IV responses/total number × 100%.

The main endpoints of the study were the development of KMP and Grade IV improvement. The secondary endpoints were drug safety and tolerance. Possible adverse reactions include diarrhea, sleep disorder, bradycardia, a drop in blood pressure, hypoglycemia, myocardial damage, and increases in liver enzymes.

### Statistical Methods

The data were processed using SPSS22.0 software. Measurement data were analyzed by descriptive statistics and are presented as mean, median, standard deviation, minimum, and maximum. Numerical data are presented as frequency (composition ratio).

## Results

### General

The study cohort comprised nine boys and two girls. The age of initiation of propranolol treatment was within 3 months in six children and within 3–6 months in three children, accounting for 81.8% of the total number. The duration of treatment ranged from 6 to 50 months (median 19.3 months). Four participants (36.3%) had a rash when born, six developed skin lesions within 3 months of birth, and the remaining one developed them 1 year after birth. Five of the participants had pain (45.4%), two had dysfunction (18.2%), and four had hyperhidrosis (36.3%). The lesions invaded skin and soft tissue in seven of the children, the muscular layer in two cases, and bone in two cases. The tumors were in the face and neck in three cases (27.3%), ear in one (9.1%), shoulder and back in two (18.2%), left thigh and hip in three (27.3%), and hand and foot in two (18.2%). The tumors grew rapidly in six cases (54.5%) and slowly in four (9.1%).

### Efficacy

The intervals between commencing oral propranolol treatment and achieving detectable improvement were 1, 2, and 3 months in each of three children in Group A, whereas it was 1 month in four children in Group B.

The treatment was effective in three of the six children in Group A, Grade I, Grade II, and Grade IV improvement, each being achieved in one child. The average duration of treatment was 7.7 months. Treatment was effective in four of the five children in Group B (two achieved Grade III improvement and two Grade IV), and the average duration of treatment was 30.8 months. The rate of effectiveness of oral propranolol against KHE was 50 and 80% in Groups A and B, respectively; this difference is significant (*P* < 0.01) (Tables 1, 2).

**Table 1:** Analysis of the therapeutic effect in each treatment group.

**Course of treatment**	**Number of cases**	**Grade of curative effect (example)**				**Efficiency (%)**
		**Grade I**	**Grade II**	**Grade III**	**Grade IV**	
Group A	6	1	1		1	50%
Group B	5			2	2	80%

**Table 2:** Clinical characteristics, treatment, and outcomes of all participants.

**Case No**.	**Onset age (m)**	**Treatment age**	**Group**	**Sex**	**Location**	**Tumor area (cm^**2**^)**	**Dysfunction**	**KMP**	**Treatment time (m)**	**Curative** **effect**
Case 1	1	2	A	Female	Right cheek	17.2	N	N	30	II
Case 2	2	2	A	Male	Left lower limb	50	N	N	12	0
Case 3	0	6	A	Male	Left hand	16.38	Y	N	10	II
Case 4	2	5	A	Male	Right shoulder, back, armpit	52	N	Y	10	I
Case 5	0	2	A	Male	Left ear	8	N	N	7	IV
Case 6	0	5	A	Male	Left thigh	195	N	N	6	I
Case 7	2	12	B	Male	Left shoulder	42	Y	N	50	IV
Case 8	0	3	B	Male	Right ankle	21	Y	N	26	IV
Case 9	13	27	B	Female	Left hip, thigh	80	N	N	34	III
Case 10	0	2	B	Male	Left face and neck	20	N	Y	15	0
Case 11	1	1	B	Male	Root of nose	26.88	N	N	13	III

*(1) Age of onset, 0 means onset immediately after birth; (2) KMP, N indicates that KMP did not develop during treatment; KMP, Y indicates that it did; (3) curative effect, 0 means ineffective*.

### Complications Developing During Treatment

KMP developed during treatment in two children, one of whom had severe thrombocytopenia. Both of these children had a history of viral infection with fever before the onset of KMP.

### Incidence of Adverse Reactions

Eight of the 11 participants (72.7%) had adverse reactions, including diarrhea, changes in ECG waveforms, left ventricular high voltage, prolonged P–R interval, decreased heart rate, and increased myocardial enzymes.

## Discussion

KHE is a rare borderline vascular tumor with a high incidence in infants and children. Severe cases of KHE are often complicated by KMP, which can lead to high mortality (6). The histological findings of KHE may overlap with those of other vascular diseases. A combination of immunohistochemical and imaging findings is helpful in distinguishing KHE from other vascular diseases (7). At present, recommended treatment varies in response to developments in the treatment of other vascular diseases and the severity of KHE. Despite many treatments being available, none of them is completely effective (8). No evidence-based, standard treatment has yet been established. Sirolimus is reportedly more effective than propranolol against KHE. However, normal vaccination schedules cannot be administered during sirolimus treatment, which is very undesirable in children aged under 1 year. Additionally, the risk of infection increases during sirolimus treatment. In contrast, propranolol treatment does not affect vaccination schedules and is extremely safe. The successful treatment of patients with relatively minor KHE skin lesions has been reported, parents being willing to try this less toxic form of treatment.

Our analysis of cases in whom treatment was ineffective revealed the following two explanations for its lack of efficacy: (1) the children had severe lesions together with the Carmel phenomenon, and (2) the dose of propranolol was too low (<1.5 mg/kg/d) and the duration of treatment too short (<1 month). To further confirm these speculations, we enrolled 11 children with mild KHE lesions for a long-term study of oral treatment with different doses of propranolol, the aim being to determine the efficacy and safety of propranolol for treating KHE. The male-to-female ratio of the 11 children was 4.5 to 1, a significantly higher proportion of male participants than the male-to-female ratio of approximately 1.3 reported by others (9).

All 11 children had large, hard, purplish-red, cutaneous plaques or masses that had slightly uneven surfaces, indistinct boundaries, and infiltrative growth. The tumors were associated with pain in five cases (45.4%), dysfunction in two (18.2%), and hyperhidrosis in four (36.3%), characteristics that distinguish KHE from infantile hemangioma (10). Although infantile hemangioma is the most common benign tumor in newborns, a hard, purplish-red, cutaneous mass appearing shortly after birth and associated with pain, hyperhidrosis, or dysfunction should prompt a consideration of the possibility of KHE.

KHE lesions characteristically grow rapidly in the early stages and then tend to stabilize over time; however, they rarely resolve completely, even with treatment. These children have been treated with immunosuppressants such as glucocorticoid and sirolimus in the past, this treatment having a relatively high incidence of adverse reactions (11). Thus, the risks and benefits of treatment should be carefully evaluated in the case of children with mild KHE. After Léauté–Labrèze first reported the successful use of propranolol to treat infantile hemangioma in 2008, this drug became a first-line treatment for high-risk infantile hemangioma (12). The findings of the studies on the efficacy of propranolol in treating KHE differ. It has been reported that the therapeutic effect is positively correlated with the blood concentration of propranolol; however, the metabolism of β-blockers differs between Asian and Caucasian individuals (13, 14). In accordance with our own experience of treating childhood hemangiomas and the characteristics of the metabolism of propranolol in Chinese individuals, we decided to assess the efficacy of 1.5 vs. 2 mg/kg of propranolol daily in the present study.

In this study, oral propranolol was effective against KHE in 50 and 80% of children in Groups A and B, respectively. This difference is statistically significant (*P* < 0.01). Such differences have not been documented for infantile hemangioma, in which the rates of efficacy of different doses of propranolol reportedly do not differ significantly. It has been reported that 1.5 mg/d/kg of propranolol is ineffective against KHE. Considering the dose dependence of responses that we have reported here, we suggest that Asian children should take 2 mg/d/kg of propranolol orally.

In this study, there was one case of KMP in each of the two groups. The platelet count was a little low in one of them and extremely low in the other. Both children had a history of viral infection and fever 1 week before onset of KMP. Some scholars have reported the development of KMP after vaccination in children with KHE, suggesting that external factors may lead to the exacerbation of KHE (15). In our case series, one of the children with KMP had KHE on the shoulder and back. Increasing this child's dose to that used in Group B stabilized the condition, and the tumor was gradually resolved. In contrast, the other patient with KMP had KHE on the face, parotid area, and neck. This child's condition continued to worsen despite the administration of high-dose propranolol. Eventually, rapamycin was substituted for propranolol. These findings suggest that the face and parotid gland are high-risk sites for KHE; this possibility requires further investigation.

There was no significant difference in the incidence of adverse reactions between Groups A and B, suggesting that propranolol 2 mg/kg/d does not cause more adverse reactions than does 1 mg/kg/d. Only one of the 11 children included in this study had to stop propranolol treatment because of adverse reactions. The rest of the children's adverse reactions were not serious and resolved with symptomatic treatment. Detailed monitoring of children during treatment is helpful in early detection and treatment of adverse reactions, potentially preventing the development of serious adverse reactions.

## Conclusions

An oral dose of 2 mg/kg/d is recommended for children with KHE who require propranolol. This treatment appears to be less effective against KHE in the parotid gland than that in extremities, and children with parotid gland KHE may be at higher risk of developing KMP. During treatment, the development of viral infection or fever should prompt clinical review, including the monitoring of the platelet count, the aim being to minimize the development of KMP. Given that this is a small case series, our conclusions are speculative; further cases need to be collected in the future to confirm our findings. Additionally, KHE cannot be completely eradicated; only long-term remission is possible. Therefore, future research must investigate the efficacy of new targeted molecular therapy options.

## Data Availability Statement

The original contributions presented in the study are included in the article/supplementary material, further inquiries can be directed to the corresponding author.

## Ethics Statement

All treatment plans have been approved by the Ethics Research Association of our school. Written informed consent was obtained from the minors' legal guardian/next of kin for the publication of any potentially identifiable images or data included in this article.

## Author Contributions

LW and LL designed the subject. BZ, ZX, XH, CW, and YL collect and follow up cases. BX analyzed experimental results. LQ analyzed data. YS assist in revising manuscripts. LW, LL, and LM wrote the manuscript. All authors contributed to the article and approved the submitted version.

## Conflict of Interest

The authors declare that the research was conducted in the absence of any commercial or financial relationships that could be construed as a potential conflict of interest.

## Publisher's Note

All claims expressed in this article are solely those of the authors and do not necessarily represent those of their affiliated organizations, or those of the publisher, the editors and the reviewers. Any product that may be evaluated in this article, or claim that may be made by its manufacturer, is not guaranteed or endorsed by the publisher.
